# The Wnt Gatekeeper SFRP4 Modulates EMT, Cell Migration and Downstream Wnt Signalling in Serous Ovarian Cancer Cells

**DOI:** 10.1371/journal.pone.0054362

**Published:** 2013-01-11

**Authors:** Caroline E. Ford, Eve Jary, Sean Si Qian Ma, Sheri Nixdorf, Viola A. Heinzelmann-Schwarz, Robyn L. Ward

**Affiliations:** 1 Wnt Signalling & Metastasis Group, Lowy Cancer Research Centre, Prince of Wales Clinical School, University of New South Wales, Sydney, New South Wales, Australia; 2 Ovarian Cancer Group, Lowy Cancer Research Centre, Prince of Wales Clinical School and School of Women's and Children's Health, University of NSW, Sydney, New South Wales, Australia; University of Alabama at Birmingham, United States of America

## Abstract

Aberrant Wnt signalling is implicated in numerous human cancers, and understanding the effects of modulation of pathway members may lead to the development of novel therapeutics. Expression of secreted frizzled related protein 4 (SFRP4), an extracellular modulator of the Wnt signalling pathway, is progressively lost in more aggressive ovarian cancer phenotypes. Here we show that recombinant SFRP4 (rSFRP4) treatment of a serous ovarian cancer cell line results in inhibition of β-catenin dependent Wnt signalling as measured by TOP/FOP Wnt reporter assay and decreased transcription of Wnt target genes, Axin2, CyclinD1 and Myc. In addition, rSFRP4 treatment significantly increased the ability of ovarian cancer cells to adhere to collagen and fibronectin, and decreased their ability to migrate across an inflicted wound. We conclude that these changes in cell behaviour may be mediated via mesenchymal to epithelial transition (MET), as rSFRP4 treatment also resulted in increased expression of the epithelial marker E-cadherin, and reduced expression of Vimentin and Twist. Combined, these results indicate that modulation of a single upstream gatekeeper of Wnt signalling can have effects on downstream Wnt signalling and ovarian cancer cell behaviour, as mediated through epithelial to mesenchymal plasticity (EMP). This raises the possibility that SFRP4 may be used both diagnostically and therapeutically in epithelial ovarian cancer.

## Introduction

Epithelial ovarian cancer has the highest mortality of all female gynaecological cancers [1], [2]. Despite recent insights into the heterogeneity of this disease [3]–[6], and a debate over the cell of origin [7], [8], most epithelial ovarian cancer patients receive the same systemic treatment (Carboplatin, Paclitaxel [5]. Further research into the molecular pathways underpinning this disease is required in order to identify novel tumour-markers and targets for therapeutic intervention. One pathway which has been identified as of potential importance in ovarian cancer is the Wnt signalling pathway [9]–[11].

The Wnt signalling pathway is a crucial developmental pathway involved in differentiation, polarity, migration, invasion, adhesion and survival [12]. These same cellular processes are key components of tumourigenesis and metastasis, and hence the role of Wnt signalling in human cancer is increasingly being investigated along with therapeutic strategies to target pathway components. Dysregulation of the Wnt signalling pathway has been implicated in numerous cancers including those with high prevalence and/or poor outcomes such as colorectal, breast, ovarian, and prostate cancer (reviewed in [13]).

This multifaceted signalling network is traditionally simplified by dividing the network into canonical (β-catenin dependent) and non-canonical (β-catenin independent) pathways. Canonical Wnt signalling involves the binding of Wnt ligand to one of ten Frizzled receptors (Fzd) in the presence of a low density lipoprotein receptor related protein (LRP) co-receptor. This generates a cascade of events leading to the disassembly of the Axin/APC/GSK3β destruction complex and the stabilisation of β-catenin. Accumulation of β-catenin in the cytoplasm results in translocation to the nucleus and TCF/LEF mediated activation of target genes involved in cell differentiation and proliferation. Key downstream targets of activated canonical Wnt signalling include C-myc (*MYC*), CyclinD1 (*CCND1*) and Axin2 (*AXIN2*) [12].

The Wnt pathway is regulated at multiple levels, with the Wnt antagonists or “gatekeeper proteins” receiving attention in recent years, due to their frequent inactivation in cancer. This group of Wnt modulators includes the Dickkopf family (DKK1-4), WIF-1 and the family of secreted frizzled receptor proteins (SFRP1-5). SFRPs are soluble extracellular proteins characterized by their frizzled-like cysteine rich domain (CRD), which is thought to be responsible for their ability to modulate Wnt signalling by binding directly to Wnt ligands or Frizzled receptors. SFRPs have been shown to function as tumour suppressors in other cancer types [14]–[17]. We have previously shown that one of these gatekeepers, SFRP4, is secreted into the blood stream and progressively lost in more aggressive ovarian cancer phenotypes, such as Type II cancers [18]. Furthermore, patients lacking SFRP4 expression had a worse prognosis than those expressing SFRP4 [18].

Epithelial to mesenchymal transition (EMT) is a key developmental process that cancer cells hijack to increase their aggressiveness and invasive potential [19]. Though complex, and cell type specific, cells undergoing EMT can generally be characterised by the loss of E-cadherin and gain of Vimentin, Twist and Snail. The transitional process may also occur in the opposite direction (mesenchymal to epithelial transition (MET).) It is now understood that MET is of equal importance in organogenesis and metastasis, and that many intermediate or “metastable” cell states exist as cells transition between the two extremes [20], [21]. This dynamic process has been termed epithelial to mesenchymal plasticity, or EMP. Many signalling pathways have been linked to EMP, notably the TGF- β, Notch and Wnt pathways.

In this present study we investigate the functional effects of modulation of a key Wnt pathway gatekeeper, SFRP4 on Wnt signalling, cell behaviour and EMT. We report for the first time that re-expression of SFRP4 in an epithelial ovarian cancer cell line inhibits Wnt signalling, increases adhesion, inhibits cell migration and inhibits EMT. Since SFRP4 expression has been shown to be lost in a large majority of ovarian cancer patients [18], this raises the possibility that modulation of Wnt signalling through SFRP4 or other upstream Wnt pathway members may represent a new avenue for targeted therapy in ovarian cancer.

## Materials and Methods

### Cell culture

Human serous ovarian cancer OVCAR3 cells, obtained from ATCC (American Type Culture Collection, Manassas, VA, USA) were cultured in RPMI containing 10% fetal calf serum. Media was supplemented with penicillin/streptomycin (90 units/ml penicillin; 90 µg/ml streptomycin) and 1.8 mM GlutaMAX (Life Technologies). All cells were grown in a humidified atmosphere of 5% CO_2_, at 37°C and were demonstrated to be free of mycoplasma contamination.

### Wnt reporter assays

OVCAR3 cells were plated at a concentration of 5000 cells/well on white bottomed 96 well plates. Cells were serum starved overnight and co-transfected with 0.2 µg of either TOPflash (3× TCF4 binding sites) or FOPflash (3× mutated TCF4 binding sites) expression plasmids (Millipore, Temecula, CA, USA), and 0.1 µg pRL-TK (Renilla-TK-luciferase vector, Promega) as a control, using Lipofectamine2000. Cells were subsequently treated with increasing doses of rSFRP4 and/or recombinant Wnt3 a (rWnt3 a 0.1 µg/ml) for 48 hours prior to luciferase activities being measured using a Glomax 96 Microplate Luminometer (Turner Biosystems Instrument, Sunnyvale, CA, USA). Firefly luciferase activity was normalized for transfection efficiency by dividing by the Renilla luciferase activity. The TOP/FOP ratio was used as a measure of β-catenin driven transcription. Average activity and standard deviations were derived from octopulate transfected samples.

### Quantitative reverse-transcriptase PCR (qPCR)

Following DNAse treatment, 1 µg of total RNA was transcribed to cDNA using the Quantitect RT kit (Qiagen, Valencia, CA, USA). Controls containing RNA but without reverse transcriptase were included for all samples. qPCR was performed in triplicate on a Stratagene MxPro 3005 P machine using 25 ng of cDNA template and SYBR green/Rox master mix (Qiagen). Expression was normalised to three different housekeeping genes (SDHA, HSPCB, YWHZA) using the Vandesompele normalisation method [22].

Primer sequences used for qPCR were SFRP4: forward (F) 5′ TGTGTTACGAGTGGCG 3′, reverse (R) 5′ GGGGGATTACTACGACTG 3′, CDH1: F 5′ AGGCCAAGCAGCAGTACATT 3′ R 5′ ATTCACATCCAGCACATCCA 3′, VIM F 5′ CCAAACTTTTCCTCCCTGAACC 3′ R 5′ GTGATGCTGAGAAGTTTCGTTGA 3′, TWIST F 5′ GCCAATCAGCCACTGAAAGG 3′ R 5′ TGTTCTTATAGTTCCTCTGATTGTTACCA 3′, AXIN2 F 5′ TCAAGTGCAAACTTTCGCCAACC 3′ R 5′ TAGCCAGAACCTATGTGATAAGG 3′, MYC F 5′ CGTCTCCACACATCAGCACAA 3′, R 5′ CACTGTCCAACTTGACCCTCTTG 3′, CCND1 F 5′ GGCGGAGGAGAACAAACAGA 3′ R 5′ TGGCACAAGAGGCAACGA 3′, JNK F 5′TCTGGTATGATCCTTCTGAAGCA 3′ R 5′ TCCTCCAAGTCCATAACTTCCTT, RHOA F 5′ GGAAAGCAGGTAGAGTTGGCT 3′ R 5′ GGCTGTCGATGGAAAAACACAT 3′, RAC1 F 5′ TGTAGTCGCTTTGCCTATTGATG 3′ R 5′ CATCGTCAGCACTAGCACAGTTT 3′, PRKCA F 5′ GCTTCCAGTGCCAAGTTTGC 3′ R 5′ CATCGTCAGCACTAGCACAGTTT 3′, SDHA F 5′ CTTGAATGAGGCTGACTGTG 3′ R 5′ ATCACATAAGCTGGTCCTGT 3′, HSPCB F 5′ TCTGGGTATCGGAAAGCAAGCC 3′ R 5′ GTGCACTTCCTCAGGCATCTTG 3′, YWHZA F 5′ ACTTTTGGTACATTGTGGCTTCAA 3′ R 5′ CCGCCAGGACAAACCAGTAT 3′.

### Western Blots

Cell lysates were prepared by lysing cells in lysis buffer (50 mM Tris-HCl (pH 7.5), 1 mM EDTA, 1 mM EGTA, 1% Triton X-100, 5 mM sodium pyrophosphate, 1 mM sodium orthovanadate, 50 mM sodium fluoride, 0.27 M sucrose, 1× complete protease inhibitor (Roche, Basel, Switzerland)). Lysates were centrifuged and the supernatant was collected for protein concentration analysis using the BCA kit (Pierce, Rockford, IL, USA). Nuclear protein lysates were prepared by lysing cells in ice-cold Nuclei Buffer (10 mM Tris, pH 7.4, 10 mM NaCl, 3 mM MgCl2, 0.1 mM EDTA and 0.5% NP-40, protease inhibitors) and incubating on ice for 10 minutes. Nuclei were recovered by centrifugation at 900g for 3 min, washed in Nuclei Wash Buffer (10 mM Tris, pH 7.4, 10 mM NaCl, 3 mM MgCl2 and 0.1 mM EDTA containing protease inhibitors) and resuspended in lysis buffer. Proteins were separated using SDS–PAGE and transferred to PVDF membranes. For Western blotting, antibodies against SFRP4 (ab32784, Abcam, Cambridge, MA, USA), E-cadherin (#3195, Cell Signaling Technology, Danvers, MA, USA), Vimentin (#5741, Cell Signaling Technology), Twist (#sc15393, Santa Cruz Biotechnology, Santa Cruz, CA, USA), β-catenin (#sc7963, Santa Cruz Biotechnology), Histone H3 (#9715s, Cell Signalling) and α-Tubulin (#3873, Cell Signaling Technology) were used. Protein bands were detected and quantified using the Image Quant LAS 4000 (GE Healthcare).

### Migration assays

OVCAR3 cells were seeded onto IBIDI Culture-Inserts (IBIDI GmbH, Am Klopferspitz 19, 82152 Martinsried, Germany) and were treated for 24 hours with rSFRP4 (5 µg/ml). IBIDI inserts were removed and closure of the resulting wound was monitored over the next 48 hours. Images of the wound were captured at different time points using the Leica DMIL microscope system (Leica Microsystems, Wetzlar, Germany).

### Adhesion assays

Tissue culture plates were coated with solutions of 10 µg/ml type I collagen (Sigma, Castle Hill, Australia), 5 µg/ml fibronectin (Millipore, Bedford, MA, United States) or 3% BSA in PBS and were incubated overnight at 37°C. Plates were rinsed with 80% ethanol, incubated in 3% BSA in serum free media for 30 min at 37°C and rinsed again with PBS. Cell suspensions were added to the coated plates and allowed to adhere for 4 hours at 37°C. Plates were gently washed with PBS, fixed with 96% ethanol and stained with 0.1% crystal violet. Excess stain was removed by extensively washing plates with sterile water. After plates had dried, cells were lysed with 50% acetic acid and absorbance was read at 595 nm using a SpectraMax Plus384 Absorbance Microplate Reader (Molecular Devices, Sunnyvale, CA, USA).

### Statistical analysis

Results are expressed as mean ± standard deviation (SD). Statistics were performed using a two-tailed student's t test. *P<0.05, **P<0.01, ***P<0.001.

## Results and Discussion

### SFRP4 inhibits Wnt signalling in a serous ovarian cancer cell line

**Figure 1:**
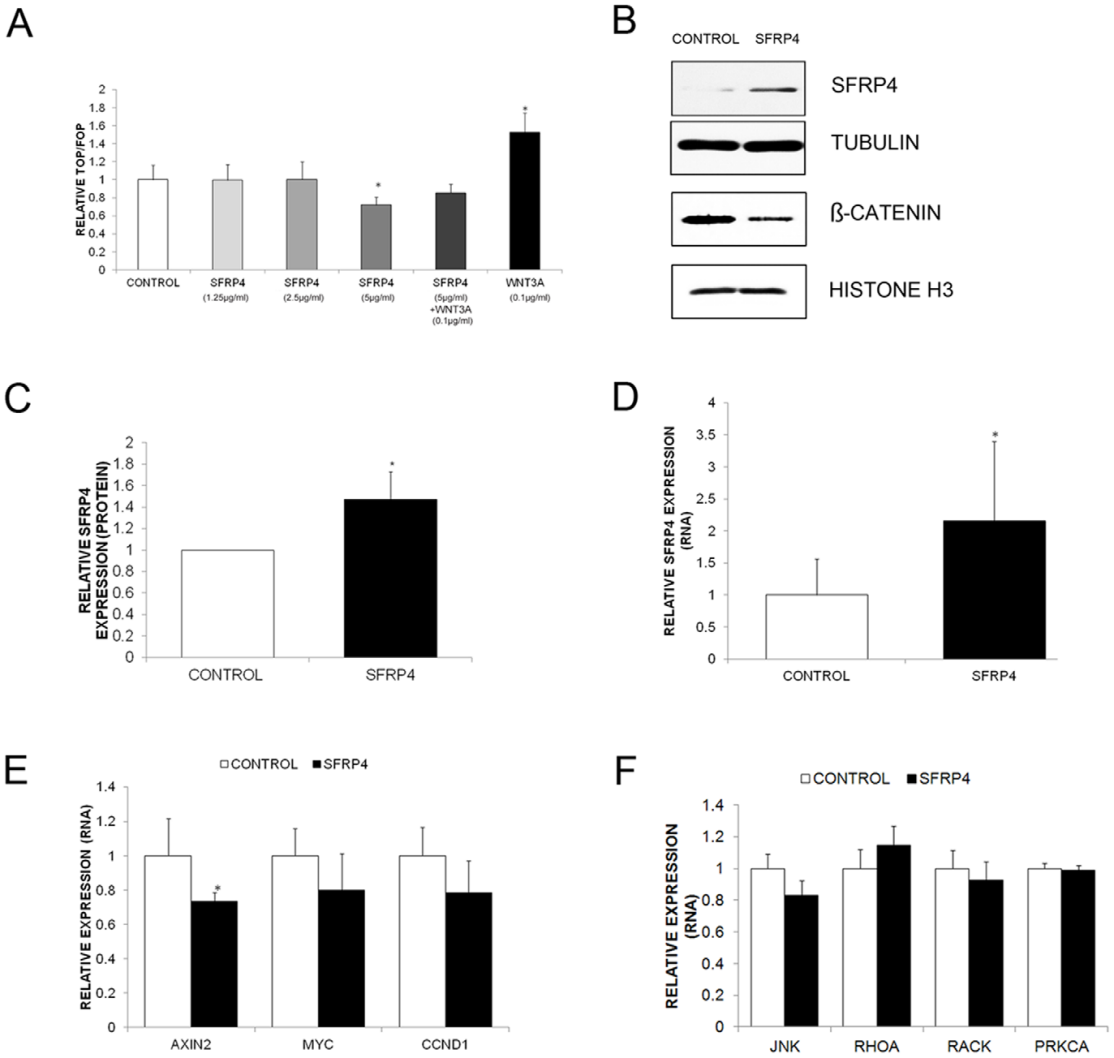
Treatment with rSFRP4 results in decreased Wnt signalling. (**A**) OVCAR3 cells were co-transfected with pRL-TK (Renilla) and either TOPflash or FOPflash expression plasmids. Cells were subsequently treated with increasing doses of rSFRP4 and/or recombinant Wnt3 a (rWnt3a 0.1 µg/ml) for 48 hours prior to luciferase activities being measured using a Glomax 96 Microplate Luminometer. Average activity and standard deviations were derived from octopulate transfected samples. Results represent the average of 3 experiments and bars represent the standard deviation (s.d) of the mean. *P<0.05. (**B**) Representative immunoblots showing increased SFRP4 and decreased nuclear β-catenin protein expression in OVCAR3 cells following 72 hours treatment with rSFRP4 (5 µg/ml). Top panel SFRP4 expression (SFRP4, ab32784, Abcam, Cambridge, UK), second panel α-tubulin expression (α-tubulin #3873, Cell Signalling Technologies, Danvers, MA, USA), third panel nuclear β-catenin (#sc7963, Santa Cruz Biotechnology), bottom panel Histone H3 (#9715s, Cell Signalling). (**C**) Densitometric analysis of SFRP4 protein expression in 3 separate experiments. Bars represent the s.d of the mean. *P<0.05. (**D**) Relative SFRP4 RNA expression was increased in OVCAR3 cells treated with rSFRP4 (5 µg/ml) for 48 hours. qRT-PCR was performed in triplicate and normalised to three different housekeeping genes (SDHA, HSPCB, YWHZA). Results represent an average of 6 experiments. Bars represent the s.d of the mean. *P<0.05. (**E**) Relative expression of β-catenin dependent Wnt target genes was decreased in OVCAR3 cells treated with rSFRP4 (5 µg/ml) for 48 hours. qRT-PCR was performed in triplicate and normalised to three different housekeeping genes (SDHA, HSPCB, YWHZA) Expression of AXIN2, MYC and CCND1 were reduced in rSFP4 treated cells, in comparison with the control group. Results represent an average of 4 experiments and bars represent the s.d of the mean. *P<0.05. (**F**) Relative expression of β-catenin independent Wnt target genes was unchanged in OVCAR3 cells treated with rSFRP4 (5 µg/ml) for 48 hours. qRT-PCR was performed in triplicate and normalised to three different housekeeping genes (SDHA, HSPCB, YWHZA) Expression of JNK, RHOA, RAC1 and PRK2A were unchanged in rSFP4 treated cells in comparison with the control group. Results represent an average of 3 experiments and bars represent the s.d of the mean.

Following on from our previous work indicating that SFRP4 loss was a frequent event in epithelial ovarian cancer [18] we determined if the addition of human recombinant SFRP4 (rSFRP4) *in vitro* could inhibit β-catenin dependent Wnt signalling. We selected the serous ovarian cancer cell line OVCAR3 for these experiments as it lacks any detectable SFRP4 expression and was previously reported to exhibit constitutive Wnt signalling (as evidenced by the accumulation/presence of nuclear β-catenin, [23]). Stimulation of OVCAR3 cells with recombinant Wnt3a (rWnt3a) resulted in a significant increase in β-catenin dependent Wnt signalling as measured via TOP/FOP FLASH luciferase Wnt reporter assay (Figure 1A), confirming that it was indeed a Wnt responsive cell line. We then tested increasing concentrations of rSFRP4, and found that 5 µg/ml of rSFRP4 was required to significantly inhibit β-catenin dependent Wnt signalling (Figure 1A). This concentration of rSFRP4 was also shown to inhibit protein levels of β-catenin in the nucleus (Figure 1B). This concentration was then used for all subsequent experiments.

Forty eight hours of treatment with rSFRP4 (5 µg/ml) lead to an approximate two fold increase in SFRP4 mRNA and protein, as the addition of extracellular recombinant Wnt proteins enhances endogenous protein production. (Figure 1B–D). The expression of three downstream Wnt target genes CyclinD1 (CCND1), C-myc (MYC) and Axin2 (AXIN2) were decreased following rSFRP4 treatment (Figure 1E). However, of these 3 genes, only AXIN2 expression was reduced significantly (*P* = 0.03). In contrast to CCND1 and MYC, AXIN2 is well accepted as a specific Wnt target gene, and to date has not been linked to other signalling pathways [24]–[27]. Increased expression of AXIN2 was reported in all serous ovarian cancer patients in a recent study [10], indicating that aberrant Wnt signalling may be more widespread in epithelial ovarian cancer than previously thought. Both MYC and CCND1 are known to function in other crucial signalling pathways implicated in ovarian carcinogenesis [28]–[31] which may explain why they exhibited weaker expression changes than AXIN2 in response to SFRP4 treatment. However, it is of note that the addition of SFRP4 in our system was capable of reducing the transcription of three downstream genes of the Wnt signalling pathway, but had no effect on downstream targets of the β-catenin independent Wnt signalling (Figure 1F) nor on an upstream receptor of the pathway (FZD7) and three unrelated receptors (EGFR, ERBB3, AKT, data not shown).

Combined, these initial experiments demonstrate that the addition of a single Wnt pathway modulator can modulate gene transcription in a model serous ovarian cancer cell line. Based on the TOP/FOP Wnt reporter assay, Western Blots and Wnt target gene results, our data also suggest that in the case of ovarian cancer, SFRP4 does indeed function as an antagonist of the canonical or β-catenin dependent Wnt signalling pathway. It also appears that SFRP4 does not act to inhibit β-catenin independent Wnt signalling, as four target genes were unaffected by treatment with SFRP4. This is of interest, as it is unclear in the field of Wnt signalling exactly which Wnt ligands are inhibited by which specific members of the SFRP family, and whether in some cases SFRPs may actually augment rather than inhibit Wnt signalling [32]–[34].

### SFRP4 treatment decreased migration and increased adhesion of ovarian cancer cells

**Figure 2:**
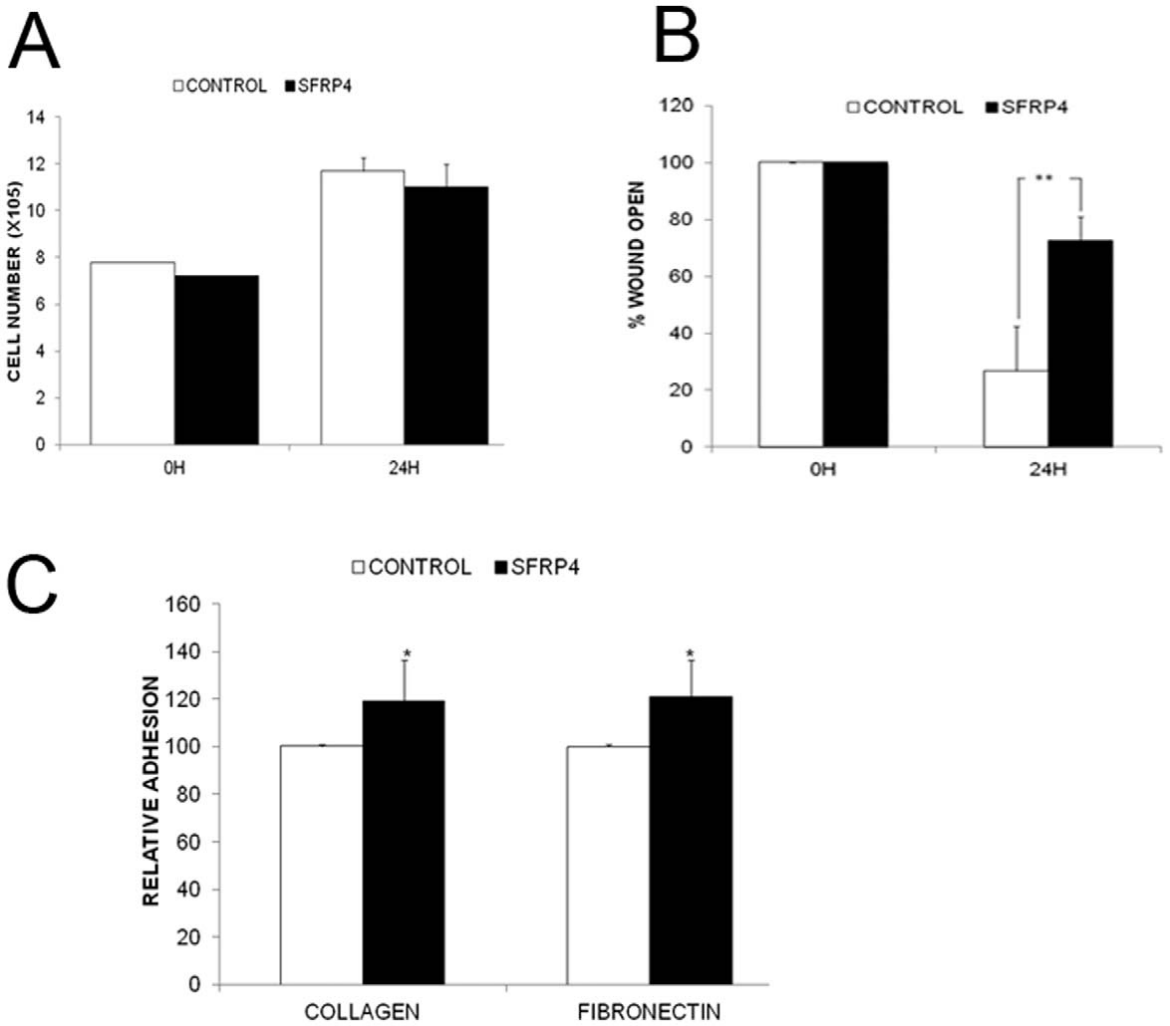
Treatment with rSFRP4 results in decreased migration and increased adhesion of ovarian cancer cells. (**A**) 24 hours rSFRP4 treatment (5 µg/ml) had no effect on cell proliferation, as measured via trypan blue cell counting using the Countess system (Invitrogen, Carlsbad, CA, USA). Results represent the average of 3 experiments and bars represent the s.d of the mean. (**B**) SFRP4 inhibited cell migration. OVCAR3 cells were seeded onto IBIDI Culture-Inserts and treated for 24 hours with rSFRP4 (5 µg/ml). IBIDI inserts were removed and closure of the resulting wound was monitored over the next 24 hours. The experiment was repeated three times and results represent the mean percentage of open wound and bars represent the s.d. **P<0.01. (**C**) SFRP4 increased adhesion to collagen and fibronectin. SFRP4 (5 µg/ml, 48 hours) treated cells were allowed to adhere for 4 hours at 37°C to either 10 ug/ml type I collagen or 5 ug/ml fibronectin, then washed, lysed and absorbance measured at 595 nm using the SpectraMax Plus384 Absorbance Microplate Reader. Results represent the average of 6 experiments and bars represent the s.d of the mean. *P<0.05.

SFRP4 treatment had no effect on cellular proliferation (Figure 2A), but significantly inhibited the ability of OVCAR3 cells to migrate across an inflicted wound (Figure 2B). It was also shown that addition of SFRP4 increased the ability of ovarian cancer cells to adhere to collagen and fibronectin coated tissue culture plates (Figure 2C). This data suggests that SFRP4 has the ability to inhibit the metastatic potential of ovarian cancer cells *in vitro*, which fits with our previous clinical data reporting that patients expressing SFRP4 had better progression free and overall survival compared with those lacking SFRP4 expression [18]. It adds to the growing body of evidence that the Wnt signalling pathway may primarily play a role in cancer metastasis rather than cancer initiation.

### SFRP4 inhibits EMT in ovarian cancer cells

**Figure 3:**
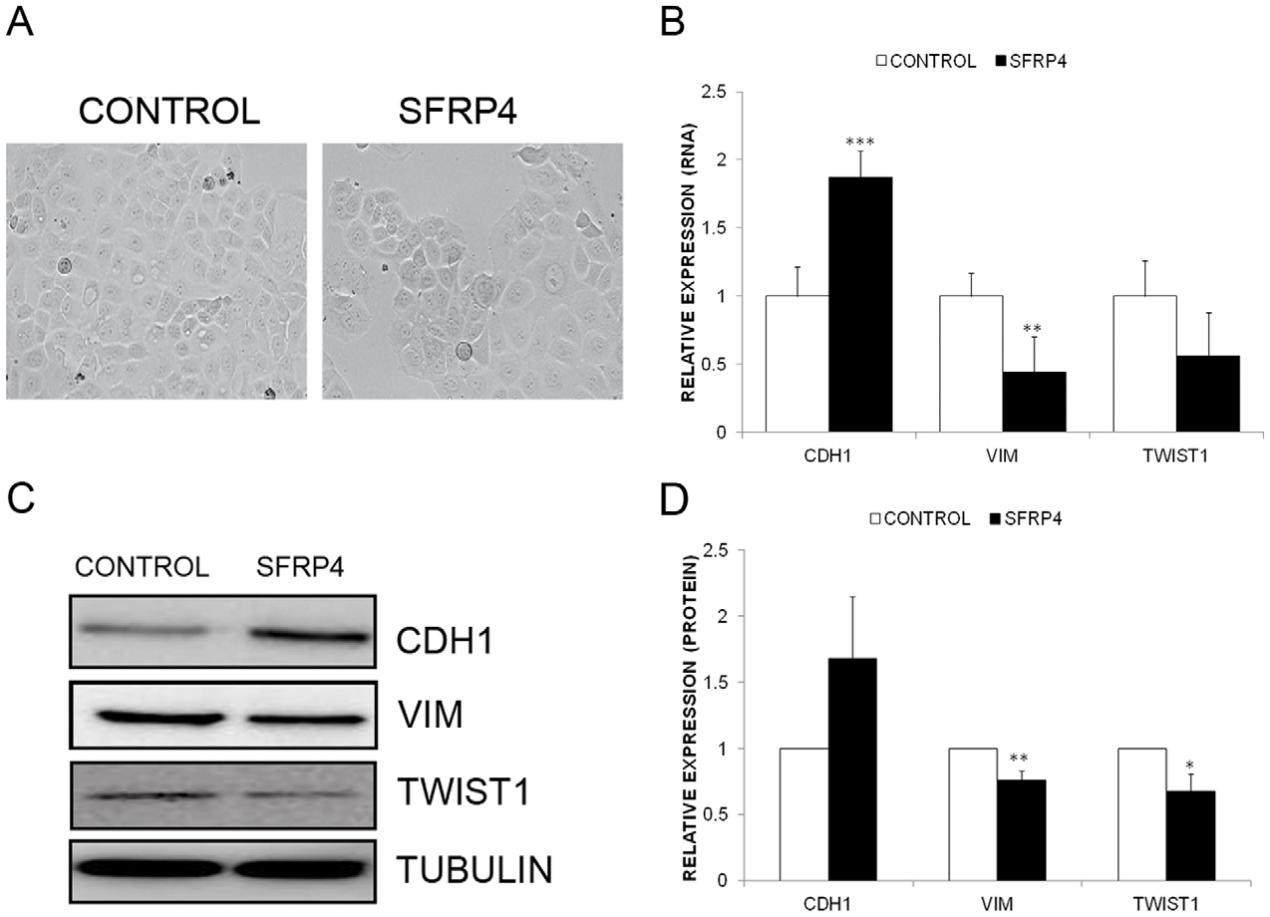
Addition of rSFRP4 results in MET in ovarian cancer cells. (**A**) SFRP4 treatment (5 µg/ml, 48 hours) did not cause any obvious changes to the cell morphology of the serous ovarian cancer cell line, OVCAR3 (10× magnification). (**B**) E-cadherin (CDH1) expression was significantly increased, and Vimentin and Twist decreased in SFRP4 (5 µg/ml, 48 hours) treated OVCAR3 cells. qRT-PCR was performed in triplicate and normalised to three different housekeeping genes (SDHA, HSPCB, YWHZA). Results represent an average of 6 experiments and bars represent the s.d of the mean. **P<0.01, ***P<0.001. (**C**) Representative immunoblots showing increased CDH1, and decreased VIM and TWIST1 protein expression in OVCAR3 cells following 72 hours treatment with rSFRP4 (5 µg/ml). Top panel CDH1 expression (#3195, Cell Signaling Technology, Danvers, MA, USA), second panel VIM expression(#5741, Cell Signaling Technology), third panel TWIST1 expression (#sc15393, Santa Cruz Biotechnology, Santa Cruz, CA, USA), and bottom panel α-tubulin expression (α-tubulin #3873, Cell Signalling Technology). (**D**) Densitometric analysis of CDH1, VIM and TWIST1 protein expression in 3 separate experiments. Bars represent the s.d of the mean. **P<0.01, *P<0.05.

As changes in cellular behaviour are linked to EMT, we also investigated whether SFRP4 would affect cell morphology and the expression of the key EMT markers, E-cadherin (CDH1), Vimentin (VIM), and Twist1 (TWIST1). While no obvious changes in cell morphology were noted 48 hours after SFRP4 treatment (Figure 3A), treatment with rSFRP4 increased the expression of E-cadherin (Figure 3B–D), and reduced expression of Vimentin and Twist (Figure 3B–D) at both the transcriptional and translational level, suggestive of initiation of MET.

EMT, like the Wnt pathway, is most strongly linked to development, yet recently has received widespread interest in its potential as a target for cancer therapy. Generalised processes that occur in all cancer cells are an attractive avenue for cancer therapy, as potential treatments will have widespread applicability. Additionally, aberrant Wnt signalling has been strongly linked to cancers with a poor prognosis [12], [35], so further elucidation of the role of this pathway and its natural gatekeepers will yield important insights into human disease. It has been known for many years that Wnt signalling can affect cancer cell migration and adhesion, and this study provides some evidence that these effects may be directed through the process of EMT. Our results lend support to a recent study linking AXIN2 and EMT in colorectal cancer [27]. In addition, we hypothesise that SFRP4 inhibits the ability of specific Wnt ligands to bind to Frizzled receptors. Based on two previous studies, one indicating that Wnt7a is overexpressed in ovarian cancer [11], and one suggesting SFRP4 can inhibit Wnt7a binding to Fzd5 in endometrial cancer [36], we speculate that under normal conditions SFRP4 binds and sequesters away Wnt7a. However in epithelial ovarian cancer, SFRP4 is silenced, allowing Wnt7a to bind Fzd5 and activate β-catenin dependent Wnt signalling, and drive TCF/LEF mediated transcription of Wnt reporter genes such as AXIN2, MYC and CCND1.

The Wnt signalling pathway is a highly complex and interconnected pathway linked to many human diseases. As the pathway is mainly involved in embryogenesis and adult tissue repair, drugs targeted to this pathway are not expected to cause significant side effects or toxicity [37]. However, attempts to target the Wnt signalling pathway in cancer have been largely unsuccessful, with no clinically approved drugs to date. This may be due to the selection of drug targets residing further downstream in the Wnt signalling pathway (recently reviewed in [38]). Numerous studies have now reported the inactivation of upstream components of the Wnt pathway in cancer including members of the SFRP and DKK families, indicating a key role for these gatekeeper proteins in oncogenesis [14]–[17], [34], [39]. These extracellular proteins also have strong potential as drug targets due to their upstream position in the pathway. This paper shows the potential of upstream pathway modulation using a single Wnt antagonist. As there are at least ten extracellular antagonists of the Wnt pathway currently identified, combinations of these may yield even stronger effects [40] and is worthy of further exploration.
